# 

**Published:** 1897-05

**Authors:** 


					﻿CONTENTS.
ORIGINAL ARTICLES—
Report of a Peculiar Case. By Joseph M. Mathews, M. D.,
Louisville, Ky........................................  .	209
Acute Tuberculosis of the Larynx. By S. G. Dabney, M. D.,
Louisville, Ky.......................................... 214
Hysterectomy for Uterine Fibromata. Fatal Termination. By
L. C. Ruter, M. D., Madison, Fla....................   217
Milk as a Source of Infection. By G. M. Corput, M. D., Atlanta.. 220
Kinks in Urine Examination.............................. 225
Remarks on Appendicitis, with a Report on Twenty-six Cases
Operated on During the Past Twelve Months. By Hunter Mc-
Guire, M. D., LL. D., Richmond, Va...........■...      226
The Bacillus of Plague.................................. 240
SELECTIONS AMD ABSTRACTS—
The Treatment of Fractures.............................. 241
Vinegar as a Hemostatic................................. 242
Diet in Dyspepsia....................................  242
Prevention and Treatment of Insanity of Pregnancy and Puer-
peral Period .....................................     244
The Surgery of the Country Practitioner............... 245
Insane Kings .	............;....................... 246
Radical Operation for Varicose Veins..............     238
Phosphuria in Diagnosis..............................  249
Horsley’s Antiseptic Wax...........................    249
The Inheritance of Mutilationsand Other Inheritances.. 250
Supposed Precocious Menstruation.....................  251
EDITORIAL.
Meeting of the Georgia Medical Association............ 252
Twelfth International Medical Congress, Moscow, Russia, August
19-26,1897.. ......................... «•........... 253
BOOK REVIEWS.........................................     254
BUSINESS DEPARTMENT...................................... 256
PRESCRIPTIONS........................................     261
				

